# Connections Between Perceived Social Support and the Body Image in the Group of Women With Diastasis Recti Abdominis

**DOI:** 10.3389/fpsyg.2021.707775

**Published:** 2021-08-09

**Authors:** Bernadetta Izydorczyk, Wiktoria Walenista, Agata Kamionka, Sebastian Lizińczyk, Magdalena Ptak

**Affiliations:** ^1^Faculty of Management and Social Communication, Institute of Applied Psychology, Jagiellonian University, Kraków, Poland; ^2^Department of Psychology, Faculty of Physical Culture, Gdansk University of Physical Education and Sport, Gdańsk, Poland; ^3^Katowice Faculty of Psychology, SWPS University of Social Sciences and Humanities, Katowice, Poland; ^4^Department of Medical Rehabilitation and Clinical Physiotherapy, Pomeranian Medical University in Szczecin, Szczecin, Poland

**Keywords:** perceived social support, body image, postpartum women, diastasis recti abdominis, childbirth

## Abstract

**Background:** The psychological features of the body image and the role of perceived social support for women with diastasis recti abdominis (DRAM) is significant for the treatment of this group of patients, but it is difficult to identify research on this topic. We aimed to search for similarities and differences between postpartum women with DRAM in terms of their psychological features of the body image and perceived social support from the partner, family and friends.

**Methods:** Three hundred forty-five Polish women with DRAM were asked to fill the The Multidimensional Body-Self Relations Questionnaire (MBSRQ), The Multidimensional Scale of Perceived Social Support (MSPSS) and The Drawing Self-Assessment Sheet. Data analysis included the stepwise regression analysis and k-cluster analysis.

**Results:** We identified several predictors in the group of women with DRAM. Social support of partner, family, and friends are the predictors of self-assessment of general body appearance. Social support of family is a predictor of self-assessment of the health of the body. Social support of friends is a predictor of self-esteem of weight and fear of gaining weight. Moreover, three clusters of women with DRAM were found. Type 1—women with DRAM with one child and low self-esteem of the general appearance of the body, low self-esteem of health condition of the body, high self-esteem of weight, and fear of weight gain, and low level of social support; Type 2—women with DRAM with three or more children and low self-esteem of the general appearance of the body, low self-esteem of health condition of the body, high self-esteem of weight and fear of weight gain, and high level of social support; and Type 3—women with DRAM with two children and high self-esteem for the general appearance of the body, high level of self-esteem for health of the body, low self-esteem of weight and fear of weight gain, and high levels of social support.

**Conclusions:** Social support is a predictor of body image in women with DRAM, but there are other factors that influence body acceptance more in this group of women. Furthermore the three clusters featured in the study may help in treating women with DRAM.

## Introduction

Research studies on the body image and its importance for psychophysical development are often described in psychological (Cash, 2004, 2017; Clark and Tiggemann, 2008; O'Dea, 2012; Dyera et al., 2013; Mantilla and Birgegård, 2015; Grogan, 2016; Izydorczyk et al., 2018, 2019, 2020; Tutkuviene et al., 2018; Alur-Gupta et al., 2019; Kertzman et al., 2019; Thomas et al., 2019; Haywood et al., 2020) and medical literature (Bolton et al., 2003; De Brito et al., 2010; Goodman et al., 2016; Anderson et al., 2017; Alur-Gupta et al., 2019; Bai et al., 2019), while in the physiotherapeutic literature, it is a rarely studied topic but important in the process of psychophysical rehabilitation of women with diastasis recti abdominis muscles (DRAM). Diastasis recti abdominis muscles is defined as a condition where both rectus abdominis muscles disintegrate to the side, being accompanied by the extension of the linea alba tissue and bulging of the abdominal wall (Michalska et al., 2018). In the field of physiotherapy, research on DRAM can be found on various health deficits, such as pain in the lumbar region (Gonçalves Fernandes da Mota et al., 2015) and perinatal abnormalities occurring during the first months after delivery (Eriksson-Crommert et al., 2020; Gustavsson and Eriksson-Crommert, 2020). However, it is difficult to find in the literature studies that verify the role of cognitive and emotional deficits in the body image in women with DRAM. A particular niche is a research on the measurement of the relationship between the influence of social standards and social support and the development of body image in women with DRAM, seen in women after childbirth. Holistic understanding of health (WHO, 2011) and approach to the human being in the state of illness and experienced deficits related to movement and psychosomatics are important for women with DRAM who require comprehensive treatment and psycho-physical rehabilitation (Gonçalves Fernandes da Mota et al., 2015; Eriksson-Crommert et al., 2020; Gustavsson and Eriksson-Crommert, 2020). The diagnosis problem of the psychological features of the body image and the role of social support for women with DRAM is a niche in scientific research, despite the fact that it is significant for the treatment and rehabilitation process of this group of patients.

Contemporary scientific research on the body image most often concerns the measurement of body image in women in the context of their body appearance after vaginal delivery (Zielinski et al., 2017), quality of sexual life (Pauls et al., 2008; Hipp et al., 2012; Thomas et al., 2019), quality of life before and after childbirth (Tutkuviene et al., 2018), body control (Hodgkinson et al., 2014; Keshwani et al., 2018, 2019; Haywood et al., 2020), social acceptance (Ogle et al., 2011; Hodgkinson et al., 2014; Keshwani et al., 2018, 2019), acceptance of their partner and family (Rallis et al., 2007; Ogle et al., 2011; Keshwani et al., 2018, 2019; Tavakoli et al., 2021), and doctor-patient relation (Keshwani et al., 2018). A review of contemporary research on the subject of the body image in the clinical group of women with DRAM confirms that studies are conducted with the participation of small groups of subjects (Ogle et al., 2011; Keshwani et al., 2018). Some of the studies available in the literature concerned women undergoing abdominoplasty (an extensive procedure aimed at eliminating the protruding abdominal fold by tensioning the transverse abdominal muscles while getting rid of excess skin and fat tissue) (Bolton et al., 2003; Keshwani et al., 2019). Research by other authors (Ogle et al., 2011; Keshwani et al., 2018) raised the context of the body image issue in a small group of women who did not undergo surgery (Ogle et al., 2011; Keshwani et al., 2018). Ogle et al. (2011) conducted a study on a group of 32 women proving that preliminary findings suggest that patients with DRAM, who were in the early postpartum phase and who were overweight or obese, had interrectus distance (IRD) negatively correlated with body image. Keshwani et al. (2018) performed the tests again on the same group of women, dividing the group of respondents into those benefiting from physiotherapy and combination therapy and patients not participating in any therapy. The positive aspect of physiotherapy influenced the body image of the patients, referring to their positive perception of their own body image. It is worth pointing out that studies on the body image by various authors were conducted using mainly clinical and various methods of body image measurement, such as questionnaires or interviews (Rallis et al., 2007; Temel et al., 2016). Health psychology, medical science, and physiotherapy indicate that the prevention and rehabilitation of women with DRAM require a holistic approach and interdisciplinary therapy (Keshwani et al., 2018). The characteristic of the symptomatology of women with DRAM is associated with the multifaceted nature of symptoms, which are associated not only with pain (Michalska et al., 2018) but also with the self-esteem of the body, emotional difficulties in accepting, and dissatisfaction with the body image (Pauls et al., 2008; Ogle et al., 2011; Hipp et al., 2012; Hodgkinson et al., 2014; Zielinski et al., 2017; Thomas et al., 2019; Aparicio et al., 2020; Haywood et al., 2020) and experienced the different quality of life after childbirth (Tutkuviene et al., 2018). Rehabilitation, such as therapeutic work, on psychological (body self-esteem, body health assessment, body weight adequacy) and social standards of posture toward the body (communicated through social support of the family) is an indispensable element of comprehensive treatment and physiotherapy of DRAM. The health service continues to enrich its experience and strategies to support women with DRAM (Gustavsson and Eriksson-Crommert, 2020). It is believed that women with these problems should go to a physiotherapist, osteopath, or doctor.

A literature review on research on the characteristic of the body image in women with DRAM confirms the difficulty in identifying research on the empirical measurement of the role of comprehensive support: partner, family, and friends; role in shaping the body image in women with DRAM. On the other hand, a significant influence can also be seen in numerous studies on sociocultural standards and family messages on the image of body and appearance in contemporary women after childbirth (Jordan et al., 2005; Coyne et al., 2018). As there are no such studies in relation to women with DRAM, we included social support (partner, family, friends) as an important variable explaining the body image in their own research. The innovation of the research in this article is also related to the measurement of intra-group similarities and differences between women with DRAM in terms of their body image, defined as a complex psychological structure including: body self-esteem (emotional dissatisfaction with the body, general cognitive acceptance of own appearance of one and acceptance of individual body parts), self-assessment of body health (assessment of the health of the body and care for the physical condition of the body), self-assessment of body weight and fear of gaining weight (fat phobia). We wanted to point out the significance of the body image features verified in the model of research of own psychological profile of one and the importance of perceived social support in the process of rehabilitation of the studied group of patients. The interdisciplinary nature of interventions in rehabilitation requires taking into account the social impact (socio-cultural and family messages) on the shaping of the body image of contemporary women. For this reason, women with DRAM, who experience real physical damage to the body, should also be treated multidimensionally. In the line of a holistic model of health, physical and psychological factors influence each other, so in this research, we wanted to check if there is such a connection between DRAM (visual change of the body that can indirectly cause pain) and the body image (a part of Self). Moreover, in this study, we used a cognitive-behavioral model of body image, because DRAM can influence the attitude toward body in a negative way. With that rationale, DRAM can be a source of body dissatisfaction, anxiety thoughts, and restrictive behavior. Interdisciplinarity in the approach to rehabilitation in women with DRAM requires social support, especially family support, which is considered as an important factor supporting treatment and physiotherapy (Jordan et al., 2005; Brytek-Matera and Rogoza, 2015). The review of contemporary literature confirms the importance of the multifaceted cognitive and emotional structure of the body image and psychosocial functioning in the treatment and rehabilitation of women with DRAM after childbirth (Pauls et al., 2008; Ogle et al., 2011; Hipp et al., 2012; Hodgkinson et al., 2014; Zielinski et al., 2017; Keshwani et al., 2018, 2019; Tutkuviene et al., 2018; Thomas et al., 2019; Haywood et al., 2020). This research is consistent with the principles of evidence-based practice in medicine, clinical psychology, and physiotherapy in women with DRAM (Jennifer et al., 2010; Blease et al., 2016).

The purpose of this article is to highlight how social support affects the body image of women with DRAM. We consider that the support of the immediate environment to be significant in the perception of the body image of women after delivery and influences other important aspects of the effectiveness of treatment and rehabilitation of DRAM after delivery. In order to verify the role of social support on body image and, thus, on supporting the treatment and rehabilitation process, we have empirically measured the relationship between perceived social support from the closest environment and body image in the population of women with DRAM after delivery. We also searched for clusters that could describe the characteristics of perceived social support and the body image in the group of women with DRAM.

## Materials and Methods

### Research Objective, Variables, and Research Questions

Building a research model in line with the research objectives and questions, we defined the dependent variable of the body image as a multi-element psychological structure that describes the evaluative and cognitive-behavioral features of the body image of women with DRAM. The variable body image contained three components: self-assessment of the general appearance of the body and its individual parts, self-assessment of the health condition of the body, and self-assessment of body weight and the level of fear of gaining weight (Cash, 2000). On the other hand, the explanatory variable was defined as perceived social support understood as a structure containing the beliefs of an individual regarding the availability of support and the possibility of using various forms of help from members of the most basic and available social support networks, that is, a partner, family, and friends (Zimet et al., 1988; Buszman and Przybyła-Basista, 2017).

The main goal of the study was to search for similarities and differences between postpartum women with DRAM in terms of their psychological features of the body image and the perception of social support from the partner, family, and friends. Also, we searched for clusters of women with DRAM, who experienced the multifactorial structure of their own body image verified in the research model, as well as an equally specific and differentiated perception of social support on the part of a partner, family, and friends.

We asked the following research questions:

- Whether and to what extent the support from the partner, family, and friends perceived by the surveyed women explains the individual psychological features of the body image identified in the research model (self-assessment of the general appearance of the body and its individual parts, self-assessment of the health condition of the body, self-assessment of the body weight, and fear of gaining weight)?
- Are there and what are the differences and similarities between women with postpartum DRAM in terms of the specific psychological features of their body image (self-assessment of the general appearance of the body and its individual parts, self-assessment of the health condition of the body, self-assessment of body weight, and the level of fear of gaining weight) and in terms of perceived social support from partner, family, and friends?

**Figure 1:**
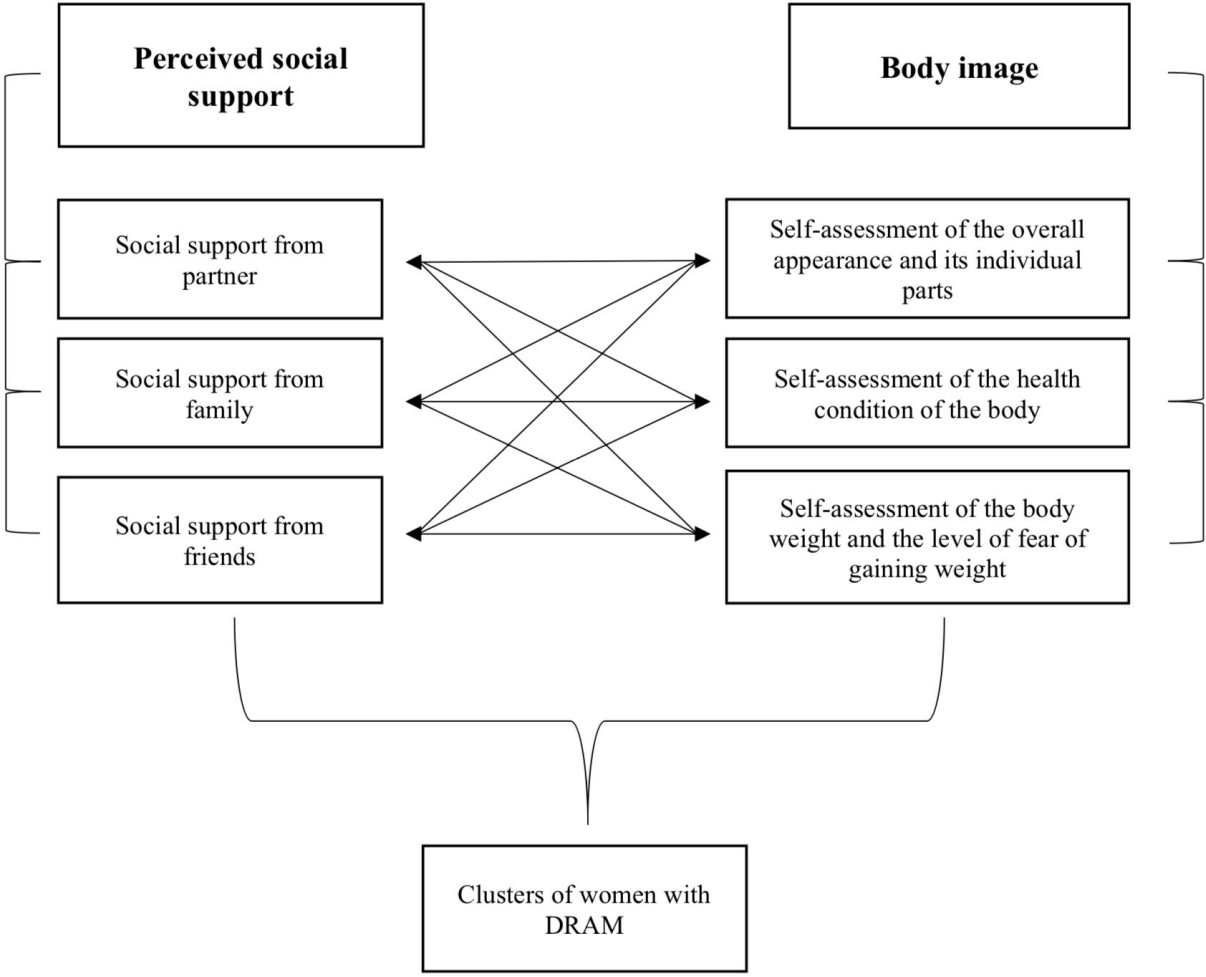
Research model of the study.

The performed statistical analyzes were primarily aimed at checking to what extent the social support perceived by women explains the evaluative and cognitive-behavioral features of the body image (self-assessment of the general appearance of the body and its individual parts, self-assessment of the health condition of the body, self-assessment of body weight and the level of fear of weight gain). Because of the fact that DRAM width is greatest immediately after delivery and can diminish in time, we decided to check these connections in three different periods after childbirth. Second, statistical analyzes were aimed at distinguishing the clusters of female body image and the characteristic of the perception of social support in women with DRAM (Figure 1).

### Ethical Approval

Ethical approval was obtained from relevant institutional ethical review committees, and the research was conducted in accordance with national and international regulations and guidelines. Written consent was obtained from all the participants. The protocol of this study was approved by the Ethics Board for Research Projects at the Institute of Applied Psychology, Jagiellonian University in Krakow.

### Participants

The selection of the group of respondents was deliberate. The criteria for inclusion and exclusion from the group of respondents were verified by survey questions regarding the presence or absence of the indicators listed below. The following inclusion criteria were used: age 20–50 years, having a confirmed medical diagnosis and participation in rehabilitation due to DRAM, having at least a child, being married, in a partner relationship or being single, Polish nationality, and living in Poland. The exclusion criteria from the group of respondents are: no children, pregnancy at the time of the study, age under 20 and over 50 years, previous abdominoplasty, physical and/or intellectual disability, and declared treatment of eating disorders. Because of the need for homogeneity in the group of women undergoing only conservative treatment, women who underwent abdominoplasty (surgical treatment) were not included in the study.

### Procedure

Initially, in the period from November 2019 to March 2020, the studies were conducted in direct contact among residents of two Polish cities in rehabilitation clinics; then, because of the epidemiological threat in Poland, the remainder of the study was conducted online from March to November 2020. The principles of the research procedure were identical for both stages of the study. The group of women with DRAM was recruited from patients of rehabilitation clinics undergoing conservative treatment and preparing for abdominoplasty surgery, and from members of support groups for women with DRAM. The purpose of the study was explained to all the surveyed women who were each time asked to give their consent to participate in the research and informed that participation in it was voluntary and anonymous. Each of the surveyed women completed a set of questionnaires and survey data (sociodemographic and medical regarding treatment) during a one-off meeting lasting from 25 to 60 min.

### Methods

The research was carried out with the use of variable measurement tools indicated below, which have high statistical accuracy and reliability:

1. The Multidimensional Body-Self Relations Questionnaire (MBSRQ) by Thomas Cash (Cash, 2017), Polish adaptation by Brytek-Matera and Rogoza (Brytek-Matera and Rogoza, 2015). The MBSRQ comprises 69 questions grouped into 10 subscales clustered into three areas: self-assessment of the general appearance of the body and its individual parts—*Appearance Evaluation* (AE), *Appearance Orientation* (AO), and *Body Areas Satisfaction* (BASS); self-assessment of the health condition of the body—*Health Evaluation* (HE) and *Health Orientation* (HO); *Illness Orientation* (IO); *Fitness Evaluation* (FE), and *Fitness Orientation* (FO); and self-assessment of body weight and the level of fear of gaining weight—*Overweight Preoccupation* (OP) and *Self-classified Weight* (SCW). The participants evaluated each item of the questionnaire by marking their answers on a five-point Likert-like scale, ranging from 1 (“definitely disagree”) to 5 (“definitely agree”). The indicators are slightly different for some items: 1 (never), 2 (rarely), 3 (sometimes), 4 (often), and 5 (very often). Furthermore, some of the items are reverse-coded. The average score for each scale should be estimated to measure the self-assessment by the respondent of their body image using the MBSRQ. In the Polish sample, the McDonald's ω ranged from 0.66 to 0.91 (Brytek-Matera and Rogoza, 2015).
2. The Multidimensional Scale of Perceived Social Support—MSPSS—is a tool developed by Zimet et al. (1988) in the Polish adaptation of Buszman and Przybyła-Basista (Buszman and Przybyła-Basista, 2017). The test consists of 12 items and has three scales—Significant Other, Family, and Friends. The respondents were asked to refer to the given statements on a seven-point scale, where 1 means “I strongly disagree” and 7—“I strongly agree.” The results can be calculated in total for the entire test or for each of the scales separately. The higher the results achieved by the respondent, the higher the level of social support they have. The MSPSS scales of the Polish version show high reliability—for the overall Cronbach's α-score it is 0.89, for the subscales: Friends −0.93, Family −0.92, and Significant Other −0.87 (Buszman and Przybyła-Basista, 2017).
3. The Drawing Self-Assessment Sheet is a projection test developed in 2011 under the leadership of K. Janowski and M. Staniewski from the University of Finance and Management in Warsaw (Błońska and Rawińska, 2015). Used in body image studies on women, the worksheet covers 25 different areas of the body and includes 50 questions. The test consists of two parts: the importance of the appearance of individual body parts and the satisfaction with their appearance. The body part appearance importance is answered on a scale from 0 to 10, where 0 means that the given body part is completely invalid, and 10—completely important. High results for this part mean that specific parts of the body are important to the individual, and low results indicate that the given part of the body is not important to them. On the other hand, the answers in the part of satisfaction with the appearance of individual body parts are also marked on a scale from 0 to 10, where 0 meant complete dissatisfaction with the appearance of a particular body part, and 10—complete satisfaction. High results for this part mean satisfaction with particular body parts. The test is not standardized, and the indicators of reliability are the average results of the first and second parts. Examples of DSAS items: 1. How important is the appearance of your hair to you?; 26. How satisfied are you with the appearance of your hair?
4. Survey with questions about sociodemographic and medical data, i.e., age, education, profession, marital status and length of last relationship, number of pregnancies and miscarriages, date of last birth, possession of DRAM now or in the past, medical diagnosis of DRAM, undergoing abdominoplasty surgery, and use of a physiotherapy treatment. The respondents also answered questions about history of mental disorders, such as eating disorders.

### Data Analysis

Statistical analyses were performed in Statistica 13.3 and in Excel (Microsoft Office 365 ProPlus).

Stages of statistical analysis:

- Stage 1—measurement of descriptive statistics. Measuring the mean values of all variables in the research model.
- Stage 2—measurement of the strength of the relationship between variables in the groups of Polish women. In this stage, Spearman's rank correlation coefficient (Spearman's rho) was used.
- Stage 3—measurement of the strength of the relationship between the dependent and independent variables by stepwise regression analysis. The aim of this stage was to search for predictors of the dependent variables in the groups of Polish women with DRAM.
- Stage 4—measurement of descriptive statistics of importance and satisfaction with the appearance of individual body parts.
- Stage 5—measurement of intragroup differences and similarities between women with DRAM—k-cluster analysis was performed for this purpose.

## Results

### Descriptive Characteristics of Medical Variables, Body Image, and Social Support in Women With DRAM

**Figure 2:**
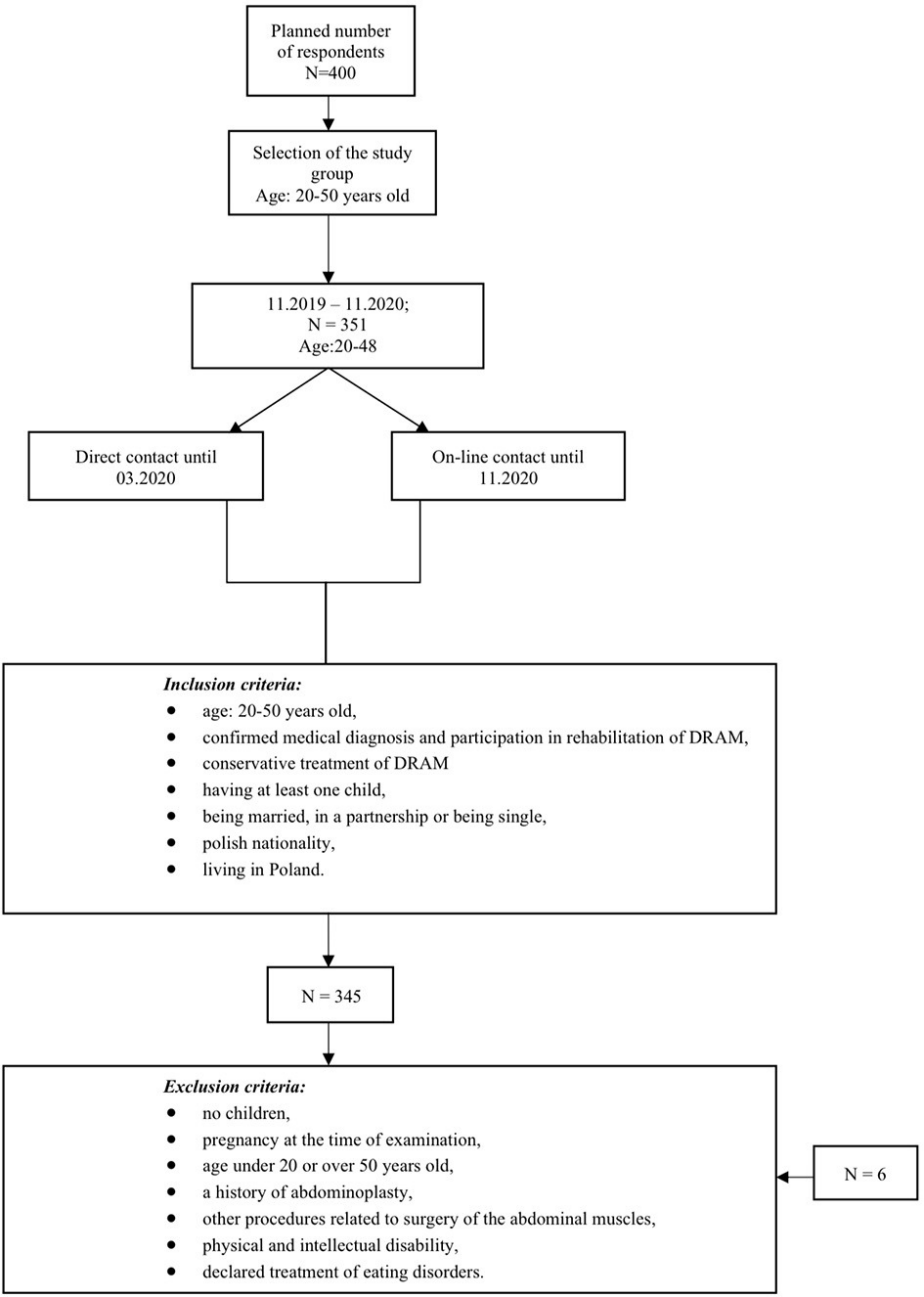
The process of selecting respondents for the research sample.

The research plan was to enroll 400 Polish women aged 20–50 years. In total, however, 351 Polish women aged 22–48 participated in the final study. Because of errors in filling in the questionnaires (undergoing abdominoplasty surgery), six women were excluded from the study (see Figure 2). The number of respondents in the sample corresponds to the size of the population of women with DRAM after delivery. The average age of the surveyed women was 32.17 years. The surveyed women had higher education (82.03%), had an administrative and office job (45.80%), were married (81.74%) and marriage lasted at least 3 years (91.28%). The respondents had two children on average, and the last childbirth took place, on average, 22.93 months ago. The mean results of social support obtained in the MSPSS test for the subscales were: Friends (FR) *M* = 22.77 (*SD* = 6.46), Family (FA) *M* = 20.57 (*SD* = 6.86), and Partner (SO) *M* = 20.42 (*SD* = 7.09). The group of surveyed women obtained lower mean results than the mean results for the Polish population in all MSPSS scales (Buszman and Przybyła-Basista, 2017). On the other hand, the mean results obtained in the MBRSQ test for individual components of the body image were: (1) self-assessment of the general appearance of the body and its individual parts—AO: *M* = 40.99 (*SD* = 6.97), AE: *M* = 20.47 (*SD* = 7.16), BASS: *M* = 27.67 (*SD* = 6.74); (2) self-assessment of the health condition of the body—HO: *M* = 26.41, (*SD* = 5.22), HE: *M* = 21.44 (*SD* = 4.54), IO: *M* = 15.49 (*SD* = 3.14), FO: *M* = 41.01 (*SD* = 10.83), FE: *M* = 9.48 (*SD* = 2.98); and (3) self-assessment of body weight and the level of fear of gaining weight—SCW: *M* = 6.54 (*SD* = 1.39), OP: *M* = 10.24 (*SD* = 3.5). The respondents obtained lower mean results in all MBRSQ subscales than the mean results for the female population (Cash, 2000) (Table 1).

**Table 1:** Descriptive characteristics of health and psychosocial variables in the studied women diagnosed with DRAM (*N* = 345).

**Variables**	***N***	***M***	***Me***	***Min***	***Max***	***SD***
Age	345	32.17	32.00	22.00	48.00	4.82
Number of children	345	1.65^*^	2.00	1.00	6.00	0.87
Months since last birth	345	22.93	20.00	1.00	70.00	16.19
Friends social support (FR)	345	22.77	25.00	4.00	28.00	6.46
Family social support (FA)	345	20.57	22.00	4.00	28.00	6.86
Partner social support (SO)	345	20.42	22.00	4.00	28.00	7.09
Self-assessment of the overall appearance and its individual parts						
Apperance orientation (AO)	345	40.99	41.00	18.00	60.00	6.97
Apperance evaluation (AE)	345	20.47	21.00	7.00	35.00	7.16
Body areas satisfaction (BASS)	345	27.67	28.00	9.00	43.00	6.74
Self-assessment of the health condition of the body						
Health orientation (HO)	345	26.41	27.00	12.00	38.00	5.22
Health evaluation (HE)	345	21.44	22.00	9.00	30.00	4.54
Illness orientation (IO)	345	15.49	16.00	9.00	25.00	3.14
Fitness orientation (FO)	345	41.01	41.00	13.00	65.00	10.83
Fitness evaluation (FE)	345	9.48	10.00	3.00	15.00	2.98
Self-assessment of the body weight and the level of fear of gaining weight						
Self-classified weight (SCW)	345	6.54	6.00	2.00	10.00	1.39
Overweight preoccupation (OP)	345	10.24	10.00	4.00	20.00	3.50

*N, number of people; M, mean; Me, median; Min, minimum value; Max, maximum value; SD, standard deviation. Self-assessment of the overall appearance and its individual parts: Appearance Evaluation (AE), assessment of satisfaction with appearance; Appearance Orientation (AO), assessment of care for appearance; Body Areas Satisfaction (BASS), assessment of satisfaction with specific body areas. Self-assessment of the health condition of the body: Health Orientation (HO), assessment of the care and commitment of a person to a healthy lifestyle; Health Evaluation (HE), self-health assessment; Illness orientation (IO), assessment of sensitivity to disease symptoms and focus on the disease; Fitness Orientation (FO), assessment of effort in building and maintaining care for one's own physical fitness; Fitness Evaluation (FE), assessment of one's own physical fitness. Self-assessment of body weight and level of fear of gaining weight: Self-Classified Weight (SCW), assessment of own body weight from underweight to overweight, assessment of where a person places themselves on the underweight-obesity scale, and their beliefs about how they would rate their weight others; Overweight Preoccupation (OP), preoccupation with being overweight and assessing the level of fear of gaining weight, the frequency of monitoring your own weight (weight vigilance), the use of various diets and weight loss*.

* *Mean values for the variable “Number of children” held are arithmetic means. In the statistical analysis of the data of these variables, the median value, which is an integer, was taken into account*.

### Characteristics of the Correlation Between Body Image Indices and Social Support

In order to statistically assess the strength of the relationship between body image indices and perceived social support, a correlation analysis (rho-Spearman's correlation coefficient) was performed. During the correlation analysis, the subjects were divided into groups according to the number of months since their last delivery. Three groups of respondents with DRAM were distinguished: women in the period 1–12 months after childbirth, women in the period 13–24 months after childbirth, and women in the period 25 months or more after childbirth. We made the following division because of the extensive research group. Moreover, body image and perceived social support can differ in motherhood stages. Data are presented in Table 2.

**Table 2:** Results of the correlation analysis (Spearman's rho) for women with DRAM in the 1–12 months postpartum period (*N* = 123).

	**AO**	**AE**	**BASS**	**FO**	**FE**	**HE**	**HO**	**IO**	**OP**	**SCW**
SO	−0.034	**0.287** ^*^	**0.459** ^*^	0.037	**0.201** ^*^	**0.246** ^*^	0.103	**0.180** ^*^	**−0.188** ^*^	−0.048
	*p* = 0.713	***p*** **=** **0.001**	***p*** **=** **0.000**	*P* = 0.686	***p*** **=** **0.026**	***p*** **=** **0.006**	*P* = 0.257	***p*** **=** **0.046**	***p*** **=** **0.037**	*p* = 0.596
FA	−0.048	**0.333** ^*^	**0.423** ^*^	**0.215** ^*^	**0.224** ^*^	**0.291** ^*^	**0.253** ^*^	**0.290** ^*^	**−0.228** ^*^	−0.138
	*p* = 0.596	***p*** **=** **0.000**	***p*** **=** **0.000**	***p*** **=** **0.017**	***p*** **=** **0.013**	***p*** **=** **0.001**	***p*** **=** **0.005**	***p*** **=** **0.001**	***p*** **=** **0.011**	*p* = 0.127
FR	−0.140	**0.301** ^*^	**0.422** ^*^	0.171	0.106	**0.308** ^*^	0.158	**0.214** ^*^	**−0.264** ^*^	−0.140
	*p* = 0.123	***p*** **=** **0.001**	***p*** **=** **0.000**	*p* = 0.059	*p* = 0.244	***p*** **=** **0.001**	*p* = 0.082	***p*** **=** **0.017**	***p*** **=** **0.003**	*p* = 0.123

* * p < 0.05. Self-assessment of the overall appearance and its individual parts: Appearance Evaluation (AE), assessment of satisfaction with appearance; Appearance Orientation (AO), assessment of care for appearance; Body Areas Satisfaction (BASS), assessment of satisfaction with specific body areas. Self-assessment of the health condition of the body: Health Orientation (HO), assessment of the care and commitment of a person to a healthy lifestyle; Health Evaluation (HE), self-health assessment; Illness orientation (IO), assessment of sensitivity to disease symptoms and focus on the disease; Fitness Orientation (FO), assessment of effort in building and maintaining care for one's own physical fitness; Fitness Evaluation (FE), assessment of one's own physical fitness. Self-assessment of body weight and level of fear of gaining weight: Self-Classified Weight (SCW), assessment of own body weight from underweight to overweight, assessment of where a person places themselves on the underweight-obesity scale, and their beliefs about how they would rate their weight others; Overweight Preoccupation (OP), preoccupation with being overweight and assessing the level of fear of gaining weight, the frequency of monitoring your own weight (weight vigilance), the use of various diets and dieting; SO, social support from the partner; FA, social support from the family; FR, social support from friends. Bold values means statistically significant*.

In the group of women with DRAM in the 1–12 months after the delivery period, the analysis of correlation coefficients showed the existence of the largest number of significant correlations of moderate or low strength between body image indices and perceived social support among the three study groups. The most significant correlations with a positive direction were shown between family social support (FA) and the assessment of care for own physical fitness of one (FO), the assessment of care for a healthy lifestyle (HO), the assessment of sensitivity to disease symptoms and focus on the disease (IO), self-health (HE), physical fitness (FE) satisfaction, visual satisfaction (AE), and BASS. Moreover, family support was negatively correlated with preoccupation with being overweight and fear of gaining weight (OP). Moreover, the existence of statistically significant correlations was shown with a positive direction between social support on the part of the partner (SO) and the assessment of sensitivity to disease symptoms and focus on the disease (IO), assessment of own health (HE), assessment of satisfaction with the level of physical fitness (FE), assessment of satisfaction with appearance (AE), and assessment of satisfaction with individual body areas (BASS). Moreover, support from the partner was negatively correlated with preoccupation with being overweight and fear of gaining weight (OP). Additionally, the existence of significant correlations was shown with a positive direction between social support from friends (FR) and the assessment of sensitivity to disease symptoms and focus on the disease (IO), assessment of own health (HE), assessment of satisfaction with appearance (AE), assessment of satisfaction with individual body areas (BASS). Social support from friends was also negatively correlated with preoccupation with being overweight and fear of gaining weight (OP).

In the group of women with DRAM in the period of 13–24 months after delivery, the analysis of correlation coefficients showed the existence of significant correlations of average or weak strength between the body image indices and the perceived social support. The most significant correlations with a positive direction were shown between family social support (FA) and the assessment of care for one's own physical fitness (FO), the assessment of care for a healthy lifestyle (HO), the assessment of sensitivity to disease symptoms and focus on the disease (IO), self-assessment of health (HE), assessment of satisfaction with appearance (AE), and assessment of satisfaction with the level of physical fitness (FE). Moreover, the existence of statistically significant correlations was shown with a positive direction between social support from the partner (SO) and the assessment of care in leading a healthy lifestyle (HO), assessment of sensitivity to disease symptoms and focus on the disease (IO), assessment of satisfaction with appearance (AE), self-assessment of health (HE) and satisfaction with the level of physical fitness (FE). Additionally, the existence of statistically significant correlations was shown with a positive direction between social support from friends (FR) and the assessment of satisfaction with appearance (AE), the assessment of own health of (HE), and the assessment of satisfaction with the level of physical fitness (FE) (Table 3).

**Table 3:** Results of the correlation analysis (Spearman's rho) for women with DRAM in the 13–24 months postpartum period (*N* = 85).

	**AO**	**AE**	**BASS**	**FO**	**FE**	**HE**	**HO**	**IO**	**OP**	**SCW**
SO	0.066	**0.219** ^*^	0.160	0.189	**0.285** ^*^	**0.257** ^*^	**0.232** ^*^	**0.302** ^*^	−0.079	−0.007
	*p* = 0.547	***p*** **=** **0.044**	*p* = 0.143	*p* = 0.083	***p*** **=** **0.008**	***p*** **=** **0.018**	***p*** **=** **0.033**	***p*** **=** **0.005**	*p* = 0.472	*p* = 0.951
FA	0.032	**0.231** ^*^	0.163	**0.249** ^*^	**0.339** ^*^	**0.315** ^*^	**0.256** ^*^	**0.227** ^*^	−0.053	−0.018
	*p* = 0.773	***p*** **=** **0.033**	*p* = 0.137	***p*** **=** **0.021**	***p*** **=** **0.002**	***p*** **=** **0.003**	***p*** **=** **0.018**	***p*** **=** **0.037**	*p* = 0.633	*p* = 0.867
FR	−0.068	**0.231** ^*^	0.199	0.093	**0.237** ^*^	**0.233** ^*^	0.169	0.092	−0.166	−0.171
	*p* = 0.536	***p*** **=** **0.033**	*p* = 0.068	*p* = 0.398	***p*** **=** **0.029**	***p*** **=** **0.032**	*p* = 0.122	*p* = 0.403	*p* = 0.129	*p* = 0.117

* *p < 0.05. Self-assessment of the overall appearance and its individual parts: Appearance Evaluation (AE), assessment of satisfaction with appearance; Appearance Orientation (AO), assessment of care for appearance; Body Areas Satisfaction (BASS), assessment of satisfaction with specific body areas. Self-assessment of the health condition of the body: Health Orientation (HO), assessment of the care and commitment of a person to a healthy lifestyle; Health Evaluation (HE), self-health assessment; Illness orientation (IO), assessment of sensitivity to disease symptoms and focus on the disease; Fitness Orientation (FO), assessment of effort in building and maintaining care for one's own physical fitness; Fitness Evaluation (FE), assessment of one's own physical fitness. Self-assessment of body weight and level of fear of gaining weight: Self-Classified Weight (SCW), assessment of own body weight from underweight to overweight, assessment of where a person places themselves on the underweight-obesity scale, and their beliefs about how they would rate their weight others; Overweight Preoccupation (OP), preoccupation with being overweight and assessing the level of fear of gaining weight, the frequency of monitoring your own weight (weight vigilance), the use of various diets and dieting; SO, social support from the partner; FA, social support from the family; FR, social support from friends. Bold values means statistically significant*.

In the group of women with DRAM 25 months and more after childbirth, the analysis of correlation coefficients showed the existence of statistically significant correlations of average and weak strength between body image indices and the perceived social support. The existence of significant correlations in the positive direction between family social support (FA) and the assessment of care in leading a healthy lifestyle (HO), the assessment of one's own health (HE), and the assessment of satisfaction with individual body areas (BASS) was demonstrated. Moreover, the existence of statistically significant correlations was shown with a positive direction between social support from friends (FR) and the assessment of care in leading a healthy lifestyle (HO), the assessment of satisfaction with appearance (AE), and the assessment of satisfaction with individual body areas (BASS). Additionally, the existence of statistically significant correlations was shown with a positive direction between social support on the part of the partner (SO) and the assessment of care in leading a healthy lifestyle (HO) and the assessment of satisfaction with individual body areas (BASS) (Table 4).

**Table 4:** Results of the correlation analysis (Spearman's rho) for women with DRAM within 25 months and more after delivery (*N* = 137).

	**AO**	**AE**	**BASS**	**FO**	**FE**	**HE**	**HO**	**IO**	**OP**	**SCW**
SO	0.067	0.160	**0.305** ^*^	0.135	0.146	0.137	**0.231** ^*^	0.072	−0.002	−0.018
	*p* = 0.440	*p* = 0.062	***p*** **=** **0.000**	*p* = 0.116	*p* = 0.088	*p* = 0.111	***p*** **=** **0.007**	*p* = 0.402	*p* = 0.985	*p* = 0.834
FA	0.034	0.130	**0.279** ^*^	0.099	0.084	**0.254** ^*^	**0.270** ^*^	0.147	0.014	0.105
	*p* = 0.691	*p* = 0.129	***p*** **=** **0.001**	*p* = 0.248	*p* = 0.332	***p*** **=** **0.003**	***p*** **=** **0.001**	*p* = 0.086	*p* = 0.874	*p* = 0.221
FR	−0.066	**0.191** ^*^	**0.306** ^*^	0.107	0.146	0.130	**0.310** ^*^	0.126	−0.082	0.017
	*p* = 0.443	***p*** **=** **0.025**	***p*** **=** **0.000**	*p* = 0.214	*p* = 0.089	*p* = 0.131	***p*** **=** **0.000**	*p* = 0.144	*p* = 0.344	*p* = 0.842

* *p < 0.05. Self-assessment of the overall appearance and its individual parts: Appearance Evaluation (AE), assessment of satisfaction with appearance; Appearance Orientation (AO), assessment of care for appearance; Body Areas Satisfaction (BASS), assessment of satisfaction with specific body areas. Self-assessment of the health condition of the body: Health Orientation (HO), assessment of the care and commitment of a person to a healthy lifestyle; Health Evaluation (HE), self-health assessment; Illness orientation (IO), assessment of sensitivity to disease symptoms and focus on the disease; Fitness Orientation (FO), assessment of effort in building and maintaining care for one's own physical fitness; Fitness Evaluation (FE), assessment of one's own physical fitness. Self-assessment of body weight and level of fear of gaining weight: Self-Classified Weight (SCW), assessment of own body weight from underweight to overweight, assessment of where a person places themselves on the underweight-obesity scale, and their beliefs about how they would rate their weight others; Overweight Preoccupation (OP), preoccupation with being overweight and assessing the level of fear of gaining weight, the frequency of monitoring your own weight (weight vigilance), the use of various diets and dieting; SO, social support from the partner; FA, social support from the family; FR, social support from friends. Bold values means statistically significant*.

To sum up, the most statistically significant correlations between the body image indices and the perceived social support occur in the group of women in the period 1–12 months after delivery with DRAM. The occurrence of significant correlations decreases with the passage of time from the onset of labor. Moreover, in all groups of the studied women, the existence of at least one significant correlation was found between the perceived social support and the following components of the body image: self-assessment of the overall appearance of the body and its individual parts (AE) and self-assessment of health condition (HE and HO).

### Social Support as a Body Image Predictor

In order to estimate the predictive role of independent variables in the studied model of variables, progressive stepwise regression model was used because of the large number of potential predictors. For this analysis, it was assumed that the independent variables (social support from friends [FR], social support from the partner [SO], social support from the family [FA], number of children, age of respondents, number of months since the last birth, and number of pregnancies) can be a predictive factor for the multivariate dependent variable: body image, i.e. self-assessment of general body appearance and individual body parts (AO, AE, and BASS), self-assessment of body health (HO, HE, IO, FO, and FE), and self-assessment of body weight and the level of fear of gaining weight (SCW and OP). The results of the regression analysis are shown in Table 5.

**Table 5:** Summary of regression models for psychosocial variables and their significant predictors in the research group of women diagnosed with DRAM (*n* = 345).

**Dependent variable**	**Independent variables**
**Self-assessment of the general appearance of the body and its individual parts**	
Assessment of care for appearance (AO)	*R*^2^ = 0.036; *F*_(4, 340)_ = 3.1754; *p* < 0.013^*^ **Social support from friends (FR) Beta** **=** –**0.195** **Social support from the partner (SO) Beta=** **0.153**
Assessment of satisfaction with appearance (AE)	*R*^2^ = 0.088; *F*_(3, 341)_ = 11.058; *p* < 0.001^***^ **Social support from friends (FR) Beta** **=** **0.143** **Number of children Beta** **=** –**0.145** **Social support from the family (FA) Beta** **=** **0.154**
Assessment of satisfaction with individual body areas (BASS)	*R*^2^ = 0,136; *F*_(4, 340)_ = 13,460; *p* < 0.001^***^ **Social support from the partner (SO) Beta=** **0.154** **Social support from friends (FR) Beta** **=** **0.155**
**Self-assessment of the health condition of the body**	
Assessment of care in leading a healthy lifestyle (HO)	*R*^2^ = 0.081; *F*_(3, 341)_ = 10.131; *p* < 0.001^***^ **Social support from the family (FA) Beta** **=** **0.209**
Self health assessment (HE)	*R*^2^ = 0.100; *F*_(3, 341)_ = 12.679; *p* < 0.001^***^ **Social support from the family (FA) Beta** **=** **0.264** **Age Beta=** **0.121** **Months since last childbirth Beta** **=** –**0.120**
Assessment of sensitivity to disease symptoms and focus on the disease (IO)	*R*^2^ = 0.078; *F*_(3, 341)_ = 9.6816; *p* < 0.001^***^ **Social support from the family (FA) Beta** **=** **0.237**
Assessment of care for one's own physical fitness (FO)	*R*^2^ = 0.033; *F*_(2, 342)_ = 5.8928; *p* < 0.003^**^ **Social support from the family (FA) Beta** **=** **0.173**
Assessment of your own physical fitness (FE)	Statistically insignificant
**Self-assessment of body weight and the level of fear of gaining weight**	
Body weight assessment from very underweight to obesity (SCW)	*R*^2^ = 0.027; *F*_(3, 341)_ = 3.2230; *p* < 0.02^*^ **Number of pregnancies Beta** **=** **0.114**
Preoccupation with being overweight and fear of gaining weight (OP)	*R*^2^ = 0.028; *F*_(1, 343)_ = 9.9608; *p* < 0.001^***^ **Social support from friends (FR) Beta** **=** –**0.168**

* *p < 0.05*;

** *p < 0.01*;

*** *p < 0.001*.

In summary, regression analysis showed a significant albeit low R^2^ predictive role of social support from friends (FR), partner (SO), and family (FA) in explaining most of the verified body image indices in women after delivery with DRAM. The exception is social support from the partner (SO) and friends (FR) explaining 13.6% of the variance in the self-assessment component of general body appearance (BASS) and social support from the family (FA) explaining 10% of the variance in the self-assessment component of fitness health of the body (HE). The values of *R*^2^ coefficients show that the higher the social support from the partner and friends, the greater the satisfaction with the appearance and individual parts of the body; the higher the social support from the family, the better the respondents assess their health. The remaining *R*^2^ coefficients in terms of assessing the strength of social support prediction for explaining the attitude toward one's own body in the surveyed postpartum women with DRAM were within the range of 2.7–8.8% of the explained variance. The above results indicate that, apart from social support, there are other significant predictors explaining body attitude in women with DRAM but not included in the study model. A summary of the significant predictors identified by the regression analysis is provided in Table 6.

**Table 6:** Summary of statistically significant predictors and variables explained on the basis of regression analysis in women diagnosed with DRAM (*N* = 345).

**Predictor**	**Dependent variables**
Family Social Support (FA)	• Self-assessment of the health condition of the body—assessment of care for one's own physical fitness (FO), assessment of own health (HE), assessment of care in leading a healthy lifestyle (HO), and assessment of sensitivity to disease symptoms and focus on the disease (IO) • Self-assessment of the general appearance of the body and its individual parts—assessment of satisfaction with appearance (AE)
Social support from friends (FR)	• Self-assessment of the general appearance of the body and its individual parts—assessment of the care for appearance (AO), assessment of satisfaction with appearance (AE) and assessment of satisfaction with individual body areas (BASS) • Self-assessment of body weight and the level of fear of gaining weight—preoccupation with being overweight and fear of gaining weight (OP)
Social support from the partner	• Self-assessment of the general appearance of the body and its individual parts—assessment of the care for appearance (AO) and assessment of satisfaction with individual body areas (BASS)
Number of children	• Self-assessment of the general appearance of the body and its individual parts—assessment of satisfaction with appearance (AE)
Number of pregnancies	• Self-assessment of the health condition of the body—assessment of own health (HE)
Months since last childbirth	• Self-assessment of body weight and the level of fear of gaining weight—assessment of own body weight from very underweight to obesity (SCW)

### Descriptive Characteristics of Importance and Satisfaction With Individual Body Parts

Referring to the results of the average severity of dissatisfaction with the body image in the surveyed women and taking into account the significant predictive role of social support from the partner and friends in the assessment of body appearance and social support from the family in the assessment of health condition, we deepened the statistical analysis focused on empirical verification of the level of satisfaction and assessment of the importance of individual body parts for the surveyed women, which was assessed using the indicators of the Drawing Appearance Self-Assessment Sheet (DASAS). This is an additional refinement of the quantitative analysis performed with the MBRSQ using the projection test. It should be mentioned that in psychology, the projective method of drawing is a recognized method of qualitative measurement (Kearney and Hyle, 2004; Błońska and Rawińska, 2015). The statistical analysis included the measurement of mean values for two DASAS scales: the scale of the importance of individual body parts for the subjects and the level of satisfaction with the appearance of individual body parts. On this basis, the most significant and highly rated individual parts of the body were distinguished for the surveyed women. The results of the subjects obtained in the DASAS test are presented in Tables 7, 8.

**Table 7:** Descriptive characteristics of means in terms of importance level assessment, i.e., focus on individual parts of the body in women diagnosed with DRAM (*n* = 345).

**Variable:** **The importance of individual parts of the body**	***M***	***Me***	***Min***	***Max***	***SD***
Hair	7.88	8.00	1.00	10.00	1.97
Eyebrows	6.85	7.00	0.00	10.00	2.38
Eyelashes	7.03	7.00	0.00	10.00	2.45
Eyes	7.67	8.00	0.00	10.00	2.31
Nose	6.58	7.00	0.00	10.00	2.41
Ears	5.51	6.00	0.00	10.00	2.83
Mouth	6.86	7.00	0.00	10.00	2.39
Teeth	8.31	9.00	0.00	10.00	1.93
Neck	5.91	6.00	0.00	10.00	2.53
Cleavage	6.37	7.00	0.00	10.00	2.52
Brests	7.54	8.00	0.00	10.00	2.25
Shoulders	6.34	7.00	0.00	10.00	2.70
Navel	5.42	6.00	0.00	10.00	3.14
Belly	8.10	9.00	0.00	10.00	2.23
Genitals	6.31	7.00	0.00	10.00	2.86
Hands	7.33	8.00	0.00	10.00	2.39
Nails	7.70	8.00	0.00	10.00	2.27
Legs	7.82	8.00	0.00	10.00	2.15
Foots	6.87	7.00	0.00	10.00	2.59
Complexion	8.22	9.00	0.00	10.00	2.09
Back	6.24	7.00	0.00	10.00	2.62
Waist	7.74	8.00	0.00	10.00	2.22
Buttocks	7.89	8.00	0.00	10.00	2.20
Hips	7.66	8.00	0.00	10.00	2.17
Calves	6.80	7.00	0.00	10.00	2.48
All parts of the body	7.08	7.28	1.36	10.00	1.75

*N, number of people; M, mean; Me, median; Min, minimum value; Max, maximum value; SD, standard deviation*.

**Table 8:** Descriptive characteristics of means in terms of the level of satisfaction, i.e., satisfaction with the appearance of individual parts of the body in women diagnosed with DRAM (*N* = 345).

**Variable:** **The importance of individual parts of the body**	***M***	***Me***	***Min***	***Max***	***SD***
Hair	6.57	7.00	0.00	10.00	2.58
Eyebrows	6.92	7.00	0.00	10.00	2.24
Eyelashes	7.21	8.00	0.00	10.00	2.26
Eyes	7.95	8.00	0.00	10.00	2.08
Nose	6.43	7.00	0.00	10.00	2.74
Ears	7.79	8.00	0.00	10.00	2.39
Mouth	7.50	8.00	0.00	10.00	2.25
Teeth	6.24	7.00	0.00	10.00	2.75
Neck	7.54	8.00	0.00	10.00	2.22
Cleavage	7.43	8.00	0.00	10.00	2.33
Brests	5.75	6.00	0.00	10.00	2.87
Shoulders	6.94	7.00	0.00	10.00	2.60
Navel	5.29	6.00	0.00	10.00	3.49
Belly	2.90	2.00	0.00	10.00	3.07
Genitals	6.17	7.00	0.00	10.00	2.87
Hands	6.65	7.00	0.00	10.00	2.68
Nails	6.61	7.00	0.00	10.00	2.75
Legs	6.15	7.00	0.00	10.00	2.86
Foots	6.05	7.00	0.00	10.00	3.01
Complexion	5.82	7.00	0.00	10.00	2.90
Back	6.66	7.00	0.00	10.00	2.65
Waist	4.73	5.00	0.00	10.00	3.26
Buttocks	5.54	6.00	0.00	10.00	2.98
Hips	5.16	5.00	0.00	10.00	2.98
Calves	6.19	7.00	0.00	10.00	2.83
All parts of the body	6.33	6.56	1.16	10.00	1.74

*N, number of people; M, mean; Me, median; Min, minimum value; Max, maximum value; SD, standard deviation*.

As part of the analysis of the descriptive characteristics of DASAS, it was shown that the ratio of importance and satisfaction with the abdomen and waist differs from the ratio of importance and satisfaction for other variables. The examined women with rectus abdominis stretch had high scores for the importance of the appearance of the abdomen and waist, and low scores for the satisfaction with the appearance of these two parts of the body.

### Characteristics of Intragroup Similarities and Differences in Body Image and Perceived Social Support in Women With DRAM—Cluster Analysis

The last stage of statistical analyzes was aimed at identifying the similarities and intra-group differences between the studied women with DRAM. Because of the analysis of many variables in the study group, the variables were standardized and then subjected to cluster analysis using the k-means method. The aim of the method was to attempt to distinguish subgroups (clusters) that will allow the identification of variables that significantly differentiate the research group (Table 9).

**Table 9:** Cluster analysis using the k-means method in the group of women diagnosed with DRAM (*N* = 345).

	**Cluster 1**	**Cluster 2**	**Cluster 3**	***df***	***F***	***p***
	***N* = 64**	***N* = 132**	***N* = 149**			
Number of pregnancies	−0.237	0.152	−0.033	342	3.4	0.033^*^
Social support from the partner (SO)	−1.607	0.376	0.357	342	246.2	0.001^*^
Family Social Support (FA)	−1.389	0.221	0.401	342	138.3	0.001^*^
Social support from friends (FR)	−1.356	0.266	0.346	342	124.3	0.001^*^
**Self-assessment of the general appearance of the body and its individual parts**						
Assessment of the care of appearance (AO)	0.101	−0.006	−0.038	342	0.4	0.648
Assessment of satisfaction with appearance (AE)	−0.552	−0.600	0.769	342	140.4	0.001^*^
Assessment of satisfaction with specific body areas (BASS)	−0.702	−0.446	0.697	342	104.1	0.001^*^
**Self-assessment of the health condition of the body**						
Assessment of care in Leading a Healthy lifestyle (HO)	−0.561	−0.375	0.573	342	58.3	0.001^*^
Self health assessment (HE)	−0.552	−0.398	0.589	342	62.5	0.001^*^
Assessment of sensitivity to disease symptoms and focus on the disease (IO)	−0.400	−0.308	0.444	342	30.6	0.001^*^
Assessment of care for one's own physical fitness (FO)	−0.474	−0.478	0.627	342	73.2	0.001^*^
Assessment of your own physical fitness (FE)	−0.569	−0.420	0.616	342	70.6	0.001^*^
**Self-assessment of body weight and the level of fear of gaining weight**						
Self-assessment of body weight and the level of fear of gaining weight body weight assessment from very underweight to obesity (SCW)	0.177	0.542	−0.556	342	57.7	0.001^*^
Preoccupation with being overweight and fear of gaining weight (OP)	0.334	0.179	−0.302	342	13.4	0.001^*^

* * p < 0.05. Negative values are due to the variable standardization. Self-assessment of the overall appearance and its individual parts: Appearance Evaluation (AE), assessment of satisfaction with appearance; Appearance Orientation (AO), assessment of care for appearance; Body Areas Satisfaction (BASS), assessment of satisfaction with specific body areas. Self-assessment of the health condition of the body: Health Orientation (HO), assessment of the care and commitment of a person to a healthy lifestyle; Health Evaluation (HE), self-health assessment; Illness orientation (IO), assessment of sensitivity to disease symptoms and focus on the disease; Fitness Orientation (FO), assessment of effort in building and maintaining care for one's own physical fitness; Fitness Evaluation (FE), assessment of one's own physical fitness. Self-assessment of body weight and level of fear of gaining weight: Self-Classified Weight (SCW), assessment of own body weight from underweight to overweight, assessment of where a person places themselves on the underweight-obesity scale, and their beliefs about how they would rate their weight others; Overweight Preoccupation (OP), preoccupation with being overweight and assessing the level of fear of gaining weight, the frequency of monitoring your own weight (weight vigilance), and the use of various diets and dieting*.

**Figure 3:**
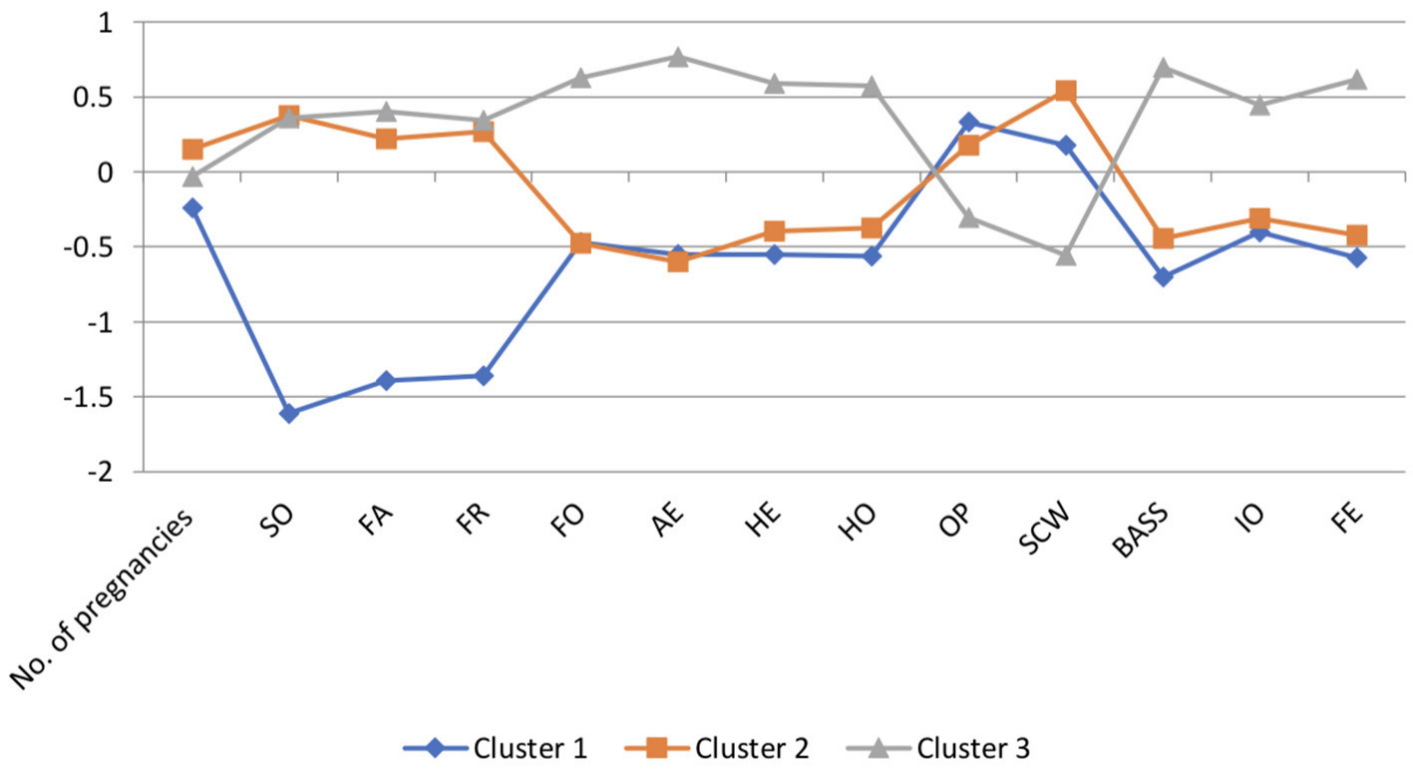
Graphic image of the selected clusters in the group of women diagnosed with DRAM (*N* = 345). Negative values are due to variable standardization. Self-assessment of the overall appearance and its individual parts: Appearance Evaluation (AE), assessment of satisfaction with appearance; Appearance Orientation (AO), assessment of care for appearance; Body Areas Satisfaction (BASS), assessment of satisfaction with specific body areas. Self-assessment of the health condition of the body: Health Orientation (HO), assessment of the care and commitment of a person to a healthy lifestyle; Health Evaluation (HE), self-health assessment; Illness orientation (IO), assessment of sensitivity to disease symptoms and focus on the disease; Fitness Orientation (FO), assessment of effort in building and maintaining care for one's own physical fitness; Fitness Evaluation (FE), assessment of one's own physical fitness. Self-assessment of body weight and level of fear of gaining weight: Self-Classified Weight (SCW), assessment of own body weight from underweight to overweight, assessment of where a person places themselves on the underweight-obesity scale, and their beliefs about how they would rate their weight others; Overweight Preoccupation (OP), preoccupation with being overweight and assessing the level of fear of gaining weight, the frequency of monitoring your own weight (weight vigilance), the use of various diets and dieting; SO, social support from the partner; FA, social support from the family; FR, social support from friends.

As a result of the conducted analysis, three significantly different clusters were distinguished (Figure 3).

### Women With DRAM Having One Child—Cluster 1

The women from Cluster 1 differ significantly in terms of the number of pregnancies and the number of children from women in Clusters 2 and 3. Cluster 1 women have a lower level of self-care (FO) and a lower level of sensitivity to disease symptoms and focus on disease (IO) compared with the women from Cluster 3. On the other hand, both women from Cluster 1 and Cluster 2 present a similar, low level of assessment of caring for physical fitness (FO) and assessment of sensitivity to disease symptoms and focus on disease (IO). Women from Cluster 1 show a lower level of satisfaction with the appearance of the body (AE) and satisfaction with the appearance of its individual parts (BASS), have a lower assessment of their own health (HE), and a lower assessment of their own physical fitness (FE) than women from Cluster 3. Compared with the women from Cluster 2, the subjects from Cluster 1 showed slight but significant differences in the assessment of satisfaction with appearance (AE) and the assessment of their own health (HE). Women from Cluster 1 assessed their weight level (SCW) as higher and showed a higher level of preoccupation with overweight and fear of gaining weight (OP) than women from Cluster 3. Among women from Cluster 1, the level of self-assessment (SCW) and preoccupation with overweight fear of weight gain (OP) was similar to that from women in Cluster 2. In terms of the perceived social support from the partner (SO), family (FA), and friends (FR), women from Cluster 1 showed significant differences with women from Clusters 2 and 3. The respondents from Cluster 1 showed a significantly lower level of perceived social support from the partner (FR), family (FA), and friends (FR) than the women in Clusters 2 and 3.

### Women With DRAM Having Two Children—Cluster 3

Women from Cluster 3 differ significantly in terms of the number of pregnancies and the number of children they have from women from Clusters 1 and 2. Cluster 3 women are characterized by a higher level of physical fitness (FO) and a lower level of sensitivity to disease symptoms and disease focus (IO) than women in the other clusters. Women from Cluster 3 show a higher level of satisfaction with appearance (AE) and its individual parts (BASS), better assess their own health (HE), and better assess their own physical fitness (FE) than women from Clusters 1 and 2. Women from Clusters 3 assessed the level of self-weight (SCW) as lower and showed lower levels of preoccupation with overweight and fear of gaining weight (OP) than women from Clusters 1 and 2. In terms of perceived social support from the partner (SO), family (FA), and friends (FR), women from Cluster 3 showed significant differences with women from Cluster 1. Women from Cluster 3 showed a significantly higher level of perceived social support from the partner (SO), family (FA), and friends (FR) than women from Cluster 1. Cluster 3 women show similar levels of social support from partner (SO), family (FA), and friends (FR) compared with Cluster 2 women.

### Women With DRAM With Three or More Children—Cluster 2

The women from Cluster 2 differ significantly in terms of the number of pregnancies and the number of children they have from the women from Clusters 1 and 3. Cluster 2 women have a lower level of physical fitness (FO) and lower sensitivity to disease symptoms and disease focus (IO) compared with Cluster 3 women. Women from Cluster 2 also show a lower level of satisfaction with appearance (AE) and its individual parts (BASS), have a lower assessment of their own health (HE), and a lower assessment of their own physical fitness (FE) than women from Cluster 3. Compared with women from Cluster 1, women from Cluster 2 showed little but significant differences in the assessment of satisfaction with appearance (AE) and the assessment of one's own health (HE). Women from Cluster 2 assess the level of self-weight assessment (SCW) as higher and show a higher level of preoccupation with being overweight and fear of gaining weight (OP) than women from Cluster 3. However, in women from Cluster 2, SCW and fear of gaining weight was at a similarly high level as for women in Cluster 1. In terms of perceived social support from partner, family, and friends, women from Cluster 2 showed significant differences with women from Cluster 1. Women from Cluster 2 showed significantly higher levels of perceived social support from partner (SO), family (FA), and friends (FR) than the women from Cluster 1. The women in Cluster 2 show a level of social support from partner, family, and friends similar to that of the women in Cluster 3.

To sum up, the cluster analysis allowed for the identification of three clusters of women, where Cluster 1 included women who present the lowest level of perceived social support from a partner (SO), family (FA), and friends (FR). In addition, women from Cluster 1 are characterized by the lowest level of concentration on physical fitness (FO) and the lowest level of sensitivity to disease symptoms (IO) among the respondents, the lowest level of physical fitness (FE), their health (HE), and the appearance of individual parts of the body (AE and BASS) are assessed the worst. Additionally, women from Cluster 1 showed the highest level of fear of gaining weight (OP) among all the respondents. In turn, women from Cluster 2 showed the lowest level of concentration on physical fitness (FO) and the worst assessment of their body appearance (AE). Moreover, the respondents from this cluster were characterized by the highest level of social support from the partner (SO) and rated their body weight as the highest among all respondents (SCW). Women from Cluster 3 rated their weight level (SCW) as the lowest and showed the lowest level of fear of gaining weight (OP). Additionally, respondents from Cluster 3 showed the highest level of social support from family (FA) and friends (FR). They rated the highest level of care for physical fitness (FO) and sensitivity to disease symptoms (IO), and showed the highest level of satisfaction with the appearance of the body (AE) and its individual parts (BASS), and the highest levels of physical fitness (FE) and health (HE) among the group of surveyed women.

## Discussion

### Perceived Social Support as a Predictor of the Body Image in Women With DRAM

The research model assumed and at the same time allowed to verify whether the multi-element variable, which is perceived social support from partner, family, and friends, and explains the multi-element structure called the body image in women with DRAM. Such model of the relationship between perceived social support and the multi-element structure of factors describing the body image (simultaneous measurement of self-assessment of the general appearance of the body and individual parts of the body, self-assessment of the health condition of the body, and self-assessment of body weight and the level of fear of gaining weight) has not been explored so widely in the earlier literature. The analysis of the results showed that the perceived social support from the partner, family, and friends significantly explains the psychological features of the body image. There are numerous statistically significant relationships between body image factors and various types of perceived social support, with the largest number of relationships reported in the group of women with DRAM in the 1–12 months postpartum period. In this group of respondents, social support from the family was most often associated with the self-assessment of the general appearance of the body and individual body parts, self-assessment of health, as well as self-assessment of weight and the level of fear of gaining weight. Additionally, the self-assessment of the general appearance of the body and individual body parts was most strongly correlated with all types of perceived social support (i.e., from partner, family, and friends). The period of the first postpartum year appears to be the most critical time for the perception of body by women with DRAM, most likely because the diagnosis and initiation of DRAM treatment usually takes place during this period. The results of this research are partially consistent with the results obtained by other authors. Bolton et al. (2003) conducted a study on 30 patients [*M* age = 37 (*SD* = 12.1); 48% married, 52% single or divorced] voluntarily undergoing abdominoplasty, where he used, inter alia, MBRSQ AO, AE, and BASS subscales. His research showed that the women surveyed before the surgery had a lower assessment of their body appearance compared with the norms for adult women; however, they were not more focused on their appearance than the normative cohort. Contrary to this study where the full version of MBRSQ was used, Bolton et al. only used three scales of this questionnaire to measure the body image. Both studies obtained similar results for the self-assessment component of general body appearance and individual body parts (AE and BASS). Although Bolton et al. did not indicate the reasons for which the respondents underwent abdominoplasty, it can be assumed that this group included women with DRAM, because this group of women willingly undergoes this procedure for both medical and aesthetic reasons. In turn, Keshwani, Mathur, and McLean (Keshwani et al., 2018) in their studies of 32 women [*M* age = 32; (*SD* = 2)] with DRAM within 3 weeks after delivery (*M* days since delivery = 22) used a shortened version of MBSRQ to test self-esteem body appearance (subscales: AE and BASS). The results of Keshwani et al. showed that the severity of symptoms of DRAM is negatively correlated with the Appearance Evaluation subscale—the greater the IRD of the subjects, the worse their body appearance was. Because of the research objectives of this study, the relationship was not studied; the relationship between the body image and DRA severity was not measured. The first year after childbirth is also the period of the most intense adaptation and acceptance of the new body, which often changes irretrievably. In the groups of women in the periods of 13–24 and 25 months after delivery, there was also a downward trend in the number of significant correlations—the more time passed since the last delivery, the less perceived social support was associated with the body image of women with DRAM. This may be due to the process of mourning after the loss of the former appearance of the body and adaptation to the altered appearance by DRAM, which is often only understood as a cosmetic defect, not a medical condition. This approach is associated with a misunderstanding on the part of the environment. The explanation for these results can be provided by the qualitative study of Eriksson-Crommert et al. (2020), who interviewed 19 women with DRAM [*M* age = 38 (*SD* = 4.8); *M* number of children = 3 (*SD* = 1.5), *M* age of the youngest child = 3 (*SD* = 1.8)]. The authors listed four groups of thematic problems reported by women with DRAM. The subjects experienced changes in the functioning of the body, especially in the abdominal area, which became a source of discomfort in everyday life. Additionally, women had difficulty accepting the change in body appearance, which was associated with lowered self-assessment. In addition, the respondents felt that they received insufficient help from specialists, and therefore had to come up with a coping strategy for DRAM themselves. In this research, we examined the same population similar to each other in terms of the age of the respondents, the number of children they have, and the time since the last delivery (time since last delivery/age of youngest child). The quantitative results presented in this article may confirm the existence of the bodily problems experienced by women with DRAM, which were illustrated in the qualitative study by Eriksson-Crommert et al. (2020). Partially, similar results were obtained by Schytt and Waldenstrom (2007), who conducted a longitudinal study on 2,424 Swedish primiparous and multiparous women [*M* age = 29.6 (*SD* = 4.6); primiparous *n* = 1,069, multiparous *n* = 1,355] regarding the risk factors of low self-assessment of health condition. Swedish studies showed that in the multiparous group, social support from a close person (2 months and one year after giving birth) and from a partner (one year after giving birth) was correlated with the risk of low self-esteem of health. We examined the self-assessment of the health condition of the body but did not conduct a longitudinal study on this variable. In this study, where the body health condition and the level of perceived social support from the partner, family, and friends were measured, the existence of statistically significant positive correlations between these variables was demonstrated.

The results presented in this article show that the body image of the subjects 1–12 months after childbirth is most sensitive to social influence and perceived social support, and seems to play an important, protective role in its creation. Nevertheless, despite the presence of a high number of statistically significant associations, their potency was weak or average, which may indicate that there are other more significant factors affecting body image that were not investigated in this study.

Regression analysis showed that perceived social support plays a statistically significant predictive role in explaining body image indices in the studied women with DRAM. Most of the *R*^2^-ratios were low, with the exception of social support from the partner explaining the BASS, and social support from family explaining the HE. These results may indicate that having a wide network of perceived social support may increase the self-assessment of the general appearance of the body and individual parts of the body, as well as the self-assessment of the health condition of the body. Low values of the *R*^2^ coefficients in terms of assessing the prediction strength of social support for explaining the body image indicate that there are other more significant predictors that were not included in the research model of this study. The above-cited Swedish research by Schytt and Waldenstrom (Zimet et al., 1988) showed that the lack of social support from the partner, relatives, and professionals, such as the nurse at the child health center, was a risk factor for low health self-esteem. We proved that perceived social support from the family was a predictor of self-assessment of the health condition of the body, which is partially consistent with the results of Swedish studies. It should be noted that, compared with the Schytt and Waldenstrom study, we performed a detailed psychometric measurement of the multifaceted variable of body image and perceived social support.

Additionally, the analysis of the importance and satisfaction of individual body parts by the DASAS test carried out in our own research showed that postpartum women with DRAM obtained high scores on the importance of the appearance of the abdomen and waist, and low scores on the scale of satisfaction with these body parts. This means that the respondents consider the appearance of the abdomen and waist to be very important, but they are dissatisfied with it, which partially confirms the results of previous studies (Bolton et al., 2003; Keshwani et al., 2018, 2019).

### Clusters of Women With DRAM

The conducted cluster analysis as well as the existing similarities and intra-group differences between women with DRAM identified allowed to distinguish three clusters. Because of the fact that the presented body image differs in these clusters and the characteristic of the perception of social support on the part of the partner, family, and friends, these types were named in the following order:

- Cluster 1—women with one child with perceived dissatisfaction with the appearance of the body, high level of fear of gaining weight, and low level of perceived social support.
- Cluster 2—women with three or more children with perceived dissatisfaction with the appearance of the body, high level of fear of gaining weight, and high level of perceived social support.
- Cluster 3—women with two children with global satisfaction with the appearance of the body without a tendency to distortions of emotional and cognitive body image and fear of gaining weight, and perceiving high social support.

On the basis of the cluster analysis, three clusters of women with DRAM were distinguished. We did not identify any studies in the literature in which the clusters of women with DRAM were measured by cluster analysis. For this reason, the research was not compared with the research of other authors in terms of distinguished clusters. On the other hand, we verified indicators of psychosocial variables (body image and perceived social support) and selected medical variables characterizing the selected clusters with the results of studies obtained by other authors.

Analyzing the results describing Cluster 1, it was shown that women with DRAM with one child have a low level of self-assessment for the general appearance of the body and its individual parts, as well as low self-assessment of body health, high self-assessment of body weight and fear of gaining weight, and low level of perceived social support from the family, partner, and friends. This is the type of women with the most negative body image and the lowest level of perceived social support among the surveyed women with DRAM. The period of pregnancy and childbirth is a borderline situation for primiparous women, changing the appearance of the body and the roles of all individuals in the family and immediate social environment of women. Because of the time required to adapt to a difficult situation, Cluster 1 women may show low levels of perceived social support from all sources closest to them and be dissatisfied with the changes in their body after giving birth. In addition, they have to deal with DRAM, the treatment of which is difficult to obtain in Poland. The research of Hung (Hung, 2007) partially confirms the results obtained by the authors of this study. In his study on a population of 861 Taiwanese primiparous and multiparous women [*M* age = 28.1 (*SD* = 4.1)], Hung showed that primiparous women assessed pregnancy-induced changes in the body more negatively than the multiparous ones. In this study, primiparous women also showed a low level of self-assessment of the general appearance of the body and its individual parts, low level of self-assessment of the health condition of the body, and high level of self-assessment of weight and fear of gaining weight. It should be noted that, compared with the Hung study, we performed a detailed psychometric measurement of a multifaceted variable of the body image. However, when it comes to comparing the perceived social support by Polish and Taiwanese primiparas, differences were shown between them. Taiwanese primiparas showed a higher level of social support from family and friends than Polish primiparas. It should be noted that Hung did not measure the social support of the partner, as was done by the authors of this study.

In contrast, Cluster 2 includes women with DRAM with three or more children who show low self-assessment of the general appearance of the body and its parts, low self-assessment of the health condition of the body, high self-assessment of body weight and fear of gaining weight but, unlike Cluster 1 women, experience a high level of social support. It seems that dissatisfaction with the body image in the case of these subjects is of different origin than in Cluster 1 women, as it may be related not so much to the lack of adaptation and acceptance of bodily changes after childbirth, but more to the accumulation of changes in body appearance due to multiple births. Cluster 2 respondents, unlike Cluster 1 women, have a high level of social support, which may be related to the fact that they are more experienced as mothers, so they and their environment had more time to adjust to the new situation such as having a baby. Probably, the multiparous women social support network has already adapted and now gives the support that are more satisfactory for mothers with three or more children. The last distinguished cluster of women with DRAM is Cluster 3, characterized by having two children, satisfaction with body appearance and health, low self-assessment of body weight and fear of gaining weight, and high level of perceived social support. Unlike the other two types, Cluster 3 women had the most positive body image, which may be because of acceptance of the changes the body goes through during pregnancy and the postpartum period and marginalizing social pressure. Moreover, the Cluster 3 respondents were characterized by a high level of social support, which, similar to Cluster 2 women, may be caused by the completion of the process of adaptation to a new life situation by the broad social environment of the surveyed women.

We used more detailed division criteria for the multiparous group than Hung, which resulted in different results for women with two children and three or more children. In a Taiwanese study, the multiparous group included women with two or more children, contrary to the division used in this study, where the multiparous group included women with two children and three or more children. In the study of Hung (2007) cited above, multiparous women assessed changes in the body after childbirth as less negative, but they perceived a lower level of social support from family and friends than the primiparas ones. The results of this study showed that two groups of multiparous women (Clusters 2 and 3) rated the level of social support from partner, family, and friends higher than that of primiparous women, contrary to the research of Hung. When it comes to body image assessment, Cluster 2 women (with three or more children) had, similar to primiparous women, a low level of self-assessment of the general appearance of the body and its individual parts, self-assessment of the health condition of the body, and a high level of self-assessment of weight and fear of gaining weight, which makes the results of this article different from those of the study of Hung. In contrast, results similar to those of the Taiwanese studies were obtained in the assessment of body image in Cluster 3 women with two children who showed a high level of self-assessment of the general appearance of the body and its individual parts, self-assessment of the health condition of the body, and a low level of self-assessment of weight and fear of gaining weight.

As part of the meta-analysis, in the study of Hodgkinson et al. (2014) on the body image in postpartum women, the theme of reclaiming the postpartum body was specified, where both primiparous and multiparous women showed a high and often unrealistic level of expectations regarding the appearance of the body after childbirth. This tendency may be reflected in the results of this study, where both Cluster 1 (primiparous women) and Cluster 2 women (multiparous women with at least three children) were less satisfied with their body appearance and body health, and assessed their weight and anxiety levels as high. The above-described division of the surveyed women with DRAM into three groups in terms of the number of children they have and the level of perceived social support may be applied in the work of physiotherapists, who are often the first professionals to whom patients with DRAM turn. Physiotherapists, being careful about the biopsychosocial context of treating somatic diseases, can pay attention to the dynamics of changes in the psychological image of the body and adapt work techniques to it. For this reason, the psychoeducation of physiotherapists seems important, as they can signal to patients with DRAM the need to seek psychological help or to strengthen existing and seek new sources of social support in the event of high levels of dissatisfaction with the appearance of the body. This holistic approach to treating patients with DRAM can speed up or improve the effects of physiotherapy.

## Limitation

The results of this research should be treated with caution, which characterizes the researcher because of the limited possibility of comparing the results with other studies in this subject area. The research procedure assumed a model of cross-sectional research, which limits the possibility of observing the dynamics of changes in the perception of body image and social support in the group of women under study over a longer period of time. Nevertheless, the obtained research results were subjected to statistical analyzes that enabled the research questions to be answered. The constructed research model, as well as the research procedure and methods, allowed us to collect an appropriate sample of respondents to make statistical analyzes appropriate to the research questions. It is also worth mentioning that the selection for the clinical group was deliberate because of the need to maintain the homogeneity of the group in terms of psychological and medical factors, e.g., mental disorders or pregnancy, which results in a limited possibility of inference on the population. In addition, selected variable measurement tools are highly valid and reliable, and are widely known and often used to measure body image and perceived social support.

A limitation in the research procedure is also the combination of online research with direct contact research, which resulted from the epidemiological situation of the ongoing coronavirus disease 2019 (COVID-19) pandemic. Nevertheless, the same research procedure was used in direct and online research.

The study did not use IRD measurements as a factor that could influence the perception of one's own body. In further research, the variable size of the DRAM should be taken into account.

## Conclusion

In summary, the obtained predictors and clusters can support a holistic approach to understanding health of women after childbirth. Because of the still low awareness of the risk of DRAM in the group of pregnant and postpartum women, these results may support the health prophylaxis of these women. First, the inclusion of psychosocial factors in the comprehensive treatment and rehabilitation of women with DRAM can affect recovery time and success.They can be a valuable clue for a physiotherapist and/or other specialist dealing with DRAM on what type of patient she is dealing with and what external factors may affect the success of therapy. Second, it can be useful for self-education of postpartum women. Moreover, the physiotherapist is often the first person to whom patients with DRAM report. Education on the perception of the body image of a woman with DRAM affects the effectiveness of physiotherapy and improves the therapeutic work of patients with DRAM with other specialists. The received holistic support of patients with DRAM may result in acceleration of the rehabilitation process and early intervention of psychological help for women. Future research on the psychological functioning of women with DRAM should focus on self and body acceptance, media influence on body perception, and perceived social support from physicians and other specialists.

## Data Availability Statement

The raw data supporting the conclusions of this article will be made available by the authors, without undue reservation.

## Ethics Statement

The studies involving human participants were reviewed and approved by Ethics Board for Research Projects at the Institute of Applied Psychology, Jagiellonian University, Krakow, Poland. The patients/participants provided their written informed consent to participate in this study.

## Author Contributions

BI, WW, and AK contributed to conception and design of the study and wrote the first draft of the manuscript. WW and SL organized the database. SL performed the statistical analysis. MP supervised the conduct of the study. All authors contributed to manuscript revision, read, and approved the submitted version.

## Conflict of Interest

The authors declare that the research was conducted in the absence of any commercial or financial relationships that could be construed as a potential conflict of interest.

## Publisher's Note

All claims expressed in this article are solely those of the authors and do not necessarily represent those of their affiliated organizations, or those of the publisher, the editors and the reviewers. Any product that may be evaluated in this article, or claim that may be made by its manufacturer, is not guaranteed or endorsed by the publisher.
